# Mesenchymal stem cell‐derived extracellular vesicles reduce senescence and extend health span in mouse models of aging

**DOI:** 10.1111/acel.13337

**Published:** 2021-03-16

**Authors:** Akaitz Dorronsoro, Fernando E. Santiago, Diego Grassi, Tianpeng Zhang, Ruenn Chai Lai, Sara J. McGowan, Luise Angelini, Mitra Lavasani, Lana Corbo, Aiping Lu, Robert W. Brooks, Marta Garcia‐Contreras, Donna B. Stolz, Antonio Amelio, Siddaraju V. Boregowda, Mohammad Fallahi, Adrian Reich, Camillo Ricordi, Donald G. Phinney, Johnny Huard, Sai Kiang Lim, Laura J. Niedernhofer, Paul D. Robbins

**Affiliations:** ^1^ Center on Aging and Departments of Molecular Medicine Scripps Research Jupiter Florida USA; ^2^ Institute on the Biology of Aging and Metabolism and Department of Biochemistry, Molecular Biology and Biophysics University of Minnesota Minneapolis Minnesota USA; ^3^ Institute of Medical Biology ASTAR Singapore Singapore; ^4^ The Shirley Ryan AbilityLab Chicago Illinois USA; ^5^ The Steadman Philippon Research Institute Vail Colorado USA; ^6^ Diabetes Research Institute University of Miami Miami Florida USA; ^7^ Department of Cell Biology University of Pittsburgh School of Medicine Pittsburgh Pennsylveniya USA; ^8^ Lineberger Cancer Center University of North Carolina Chapel Hill North Carolina USA; ^9^ Department of Bioinformatics Scripps Research Jupiter Florida USA

**Keywords:** aging, extracellular vesicles, mesenchymal stem cells, senescence, stem cells

## Abstract

Aging drives progressive loss of the ability of tissues to recover from stress, partly through loss of somatic stem cell function and increased senescent burden. We demonstrate that bone marrow‐derived mesenchymal stem cells (BM‐MSCs) rapidly senescence and become dysfunctional in culture. Injection of BM‐MSCs from young mice prolonged life span and health span, and conditioned media (CM) from young BM‐MSCs rescued the function of aged stem cells and senescent fibroblasts. Extracellular vesicles (EVs) from young BM‐MSC CM extended life span of *Ercc1* ^−/−^ mice similarly to injection of young BM‐MSCs. Finally, treatment with EVs from MSCs generated from human ES cells reduced senescence in culture and *in vivo*, and improved health span. Thus, MSC EVs represent an effective and safe approach for conferring the therapeutic effects of adult stem cells, avoiding the risks of tumor development and donor cell rejection. These results demonstrate that MSC‐derived EVs are highly effective senotherapeutics, slowing the progression of aging, and diseases driven by cellular senescence.

## Graphical abstract

Extracellular vesicles from young bone marrow‐derived mesenchymal stem cells (MSC) reduce markers of senescence in vitro. EVs derived from MSCs generated from human embryonic stem cells reduced expression of senescence markers in culture and in vivo in accelerated and naturally aged mice and improved measures of healthspan. This work demonstrates the senotherapeutic potential of extracellular vesicles in suppressing senescence‐driven age related disease.

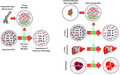

## 1 INTRODUCTION

With aging comes an inevitable and progressive loss of the ability of tissues to recover from stress. Consequently, the incidence of chronic degenerative diseases increases exponentially beginning at age 65 and is accompanied by an elevated risk for neurodegenerative diseases, cardiovascular disease, diabetes, osteoarthritis, cancers, and osteoporosis. More than 90% of people over 65 years of age have at least one chronic disease while 75% have two or more comorbidities. Thus, it is imperative to find a way to therapeutically target the cellular processes underlying aging in order to compress the period of functional decline in old age. Such a therapeutic approach would simultaneously prevent, delay, or alleviate multiple diseases of old age.

Senescence is a cell fate that involves loss of proliferative potential of normally replication‐competent cells, with associated resistance to cell death through apoptosis, and generally increased metabolic activity. Some senescent cells develop a senescence‐associated secretory phenotype (SASP) involving increased secretion of pro‐inflammatory cytokines and chemokines, tissue‐damaging proteases, and factors that can impact stem and progenitor cell function and growth factors (Tchkonia et al., 2013). Markers of senescence include elevated expression of the cyclin‐dependent kinase inhibitors p16^INK4a^ and p21^Cip1^, of SASP factors (e.g., IL‐6, IL‐1β, TNFα, and many others), increased senescence‐associated β‐galactosidase (SA‐β‐gal) activity, and telomere‐associated DNA damage foci (TAFs). In support of an important role for senescence in aging, selective ablation of p16^INK4a^‐positive senescent cells extended health span in transgenic mouse models of accelerated and natural aging (Baker et al., ,^2011^, ^2016^; Jeon et al., 2017; Kirkland & Tchkonia, 2017; Kirkland et al., 2017). Clearance of senescent cells in the INK‐ATTAC and p16‐3MR mouse genetic models or treating mice with novel senolytics extended health span (Fuhrmann‐Stroissnigg, Ling, et al., 2017; Zhu et al., 2015) and ameliorated the symptoms of numerous pathologies (Childs et al., 2016; Jeon et al., 2017; Ogrodnik et al., 2017; Roos et al., 2016; Schafer et al., 2017). Thus, the increase in cellular senescence that occurs with aging plays a major role in driving life‐limiting, age‐related diseases (Kirkland & Tchkonia, 2015, 2017; Kirkland et al., 2017; LeBrasseur et al., 2015; Palmer et al., 2015; Tchkonia et al., 2013).

A characteristic of aging is the loss of regenerative capacity, which leads to an impaired ability to respond to stress and therefore increased morbidity and mortality. This has led to the hypothesis that aging is partly driven by the loss of functional adult stem cells necessary for maintenance of tissue homeostasis. Indeed, mice greater than two years of age have a significant reduction in the number and proliferative capacity of various types of adult stem cells.

We previously demonstrated that muscle‐derived stem/progenitor cells (MDSPC) are adversely affected upon aging (Lavasani et al., 2012). MDSPCs isolated from old and *Ercc1* ^−/∆^ progeroid mice have reduced proliferative capacity and impaired differentiative potential, and this dysfunction directly contributes to age‐related degeneration given that transplantation of young MDSPCs extended health span and life span in ERCC1‐deficient progeroid mouse models. Transplanted MDSPCs did not differentiate or migrate from the site of injection, suggesting that the therapeutic effect of MDSPCs was mediated by secreted factors acting systemically (Lavasani et al., 2012). Concordantly, co‐culture of young MDSPCs with old MDSPCs resulted in renewal of old MDSPC proliferative and differentiative potential, yet the identification of factors responsible for the rejuvenation of aged MDSPCs remained elusive.

Here, we identified BM‐MSCs from young animals, and lineage‐directed hESC‐derived BM‐MSC surrogates, as a novel source of EVs with senotherapeutic activity. We demonstrate that transplantation of BM‐MSCs from young, but not old mice, prolonged life span and health span in ERCC1‐deficient mice. Further, conditioned media (CM) from young BM‐MSCs rescued the function of aged, senescent stem cells and senescent murine embryonic fibroblasts (MEFs) in culture. Moreover, the senotherapeutic activity of CM co‐purified with extracellular vesicles (EVs) that were released by young, but not old MSCs and MDSPCs. Importantly, IP injection of EVs from BM‐MSCs from young mice extended the life span of ERCC1‐deficient mice. Similarly, treatment with EVs isolated from human embryonic stem cell‐derived MSCs (hESC‐MSC) was capable of significantly reducing the expression of markers of senescence in cultured senescent fibroblasts as well as naturally aged wild‐type and *Ercc1* ^−/∆^ mice, and improving measures of healthspan *in vivo*. These novel results identified EVs as key factors released by young, functional stem cells that can rescue cellular senescence and stem cell dysfunction in culture and reduce senescent cell burden *in vivo*. Thus, functional stem cell‐derived EVs represent a novel therapeutic to reduce the senescent cell burden and extend health span.

## 2 RESULTS

### 2.1 Aged murine bone marrow‐derived mesenchymal stem cells are dysfunctional

**FIGURE 1:**
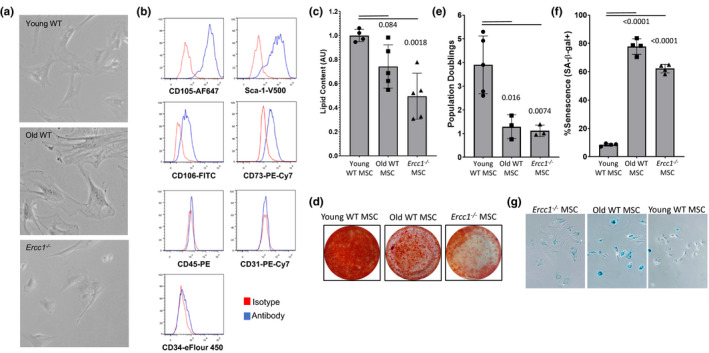
Murine bone marrow‐derived mesenchymal stem cells from naturally aged and mouse models of accelerated aging are dysfunctional. (a) Bone marrow‐derived MSCs (BM‐MSCs) from *Ercc1* ^−/−^ mice are morphologically similar to BM‐MSCs from young mice and (b) share similar phenotypic membrane markers. (c) BM‐MSCs from naturally aged and *Ercc1* ^−/−^ mice show impaired adipogenic potential. MSCs from different mouse models were differentiated to adipocytes using adipogenic media for 21 days. The lipid content was quantified using Nile Red stain and the values normalized to young WT mouse values. Bar graph shows mean ±SD from 3 independent experiments. Significance was determined by one‐way ANOVA (*p* = 0.0024, *F*(2,11) = 11.01) with Tukey's multiple comparisons test; *p* value for specific comparisons shown in figure. (d) BM‐MSCs from naturally aged and *Ercc1* ^−/−^ mice have impaired osteogenic potential. MSC from the different mouse models was differentiated to osteocytes using osteogenic media for 21 days. Calcium matrix was stained using Alizarin Red S. Photographs show a differentiation representative of 3 independent experiments. (e) Proliferative potential of old WT and *Ercc1* ^−/−^ BM‐MSCs is reduced compared to WT BM‐MSC controls. Significance was determined by one‐way ANOVA (*p* = 0.0038, *F*(2,8) = 12.11) with Tukey's multiple comparisons test; *p* values for specific comparisons shown in figure. (f) Quantitation of SA‐β‐gal staining, and representative brightfield (X‐gal) microscopy (g) of BM‐MSCs from naturally aged and *Ercc1* ^−/−^ mice, which undergo senescence earlier than BM‐MSCs from young mice. The percentage of senescent cells was manually determined by counting the percent of cells positive for senescence‐associated β‐galactosidase at passage 5. Bar graph shows mean ±SD from 3 replicate representing 3 independent experiments. Significance was determined by one‐way ANOVA (*p* < 0.0001, *F*(2,9) = 394.3) with Tukey's multiple comparisons test; *p* values for specific comparisons shown in figure

We previously demonstrated MDSPC functional decline with natural and accelerated aging in ERCC1‐deficient mice in regard to proliferation and differentiation. To determine if other types of adult stem cells have similar dysfunction, we examined the effect of age on murine bone marrow‐derived MSCs. BM‐MSC were isolated from young (3–20 weeks) and old (2 years) WT, and young *Ercc1* ^−/−^ mice (Figure 1a), and phenotyped for markers of MSCs (Figure 1b). Non‐senescent, low‐passage young WT, and *Ercc1* ^−/−^ MSCs (Figure 1a, top and bottom panels) were smaller and shared more morphological similarities than the enlarged old WT MSCs (middle). The low‐passage MSCs from old mice and *Ercc1* ^−/−^ mice have reduced adipogenic (Figure 1c) and osteogenic potential (Figure 1d). Proliferation rates of BM‐MSCs from old and *Ercc1* ^−/−^ mice were reduced with each cell passage (Figure 1e). Quantification of SA‐ß‐gal positive cells at passage 5 showed that both old WT and ERCC1‐deficient BM‐MSCs senesce more rapidly in culture, with more than 65% of cells staining positive for SA‐ß‐gal compared to less than 10% with BM‐MSC derived from young WT mice (Figure 1f and 1g). RNA‐seq analysis of young and old MSCs identified several mRNAs and miRNAs that were differentially expressed and Ingenuity Pathway Analysis (IPA) of the differentially expressed mRNAs identified key pathways affected by age (Figure S1a). The majority of these pathways also were identified by IPA analysis of the differentially expressed miRNAs (Figure S1b).

### 2.2 Treatment with young BM‐MSCs extends life span of Ercc1^−/−^ mice

**FIGURE 2:**
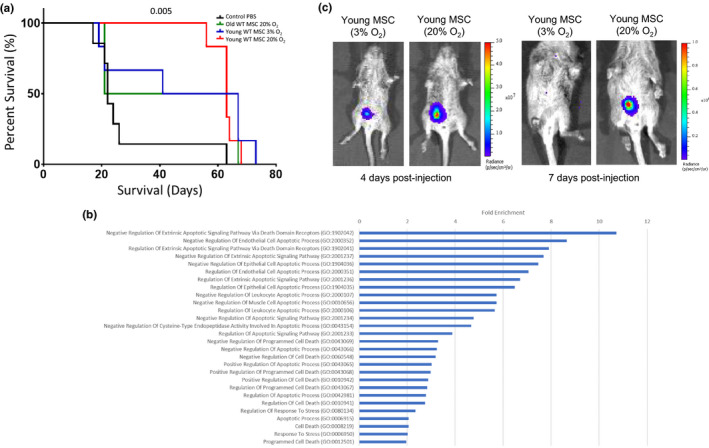
Intraperitoneal injection of young BM‐MSCs extends life span of *Ercc1* ^−/−^ mice. (a) Survival curves of *Ercc1* ^−/−^ mice treated with young and old, stressed and unstressed BM‐MSCs. Approximately 10^6^ BM‐MSCs from young and old mice were injected into *Ercc1* ^−/−^ mice by intraperitoneal injection at postnatal day 10. The BM‐MSCs from young mice were maintained at 3% O_2_ or shifted to 20% O_2_ for 48 hours prior to injection. A minimum of 4 mice per group were monitored and compared against PBS‐injected controls. Survival was compared using the log‐rank Mantel‐Cox test; *p* value for specific comparison shown in figure. (b) Gene ontology (GO; molecular function) analysis of the transcriptome of BM‐MSCs from young mice cultured for 48 h at 20% O_2_ compared to BM‐MSCs maintained at 3% O_2_. Bars are all significant post‐FDR correction (*p* < 0.05) log_2_ fold‐enrichment values for molecular function categories related to cell survival. (c) BM‐MSCs from young mice transduced with pRETOX‐TIGHT‐GpNLuc were briefly stimulated with oxidative stress, injected IP and tracked using IVIS Xenogen imager system 4 days (left panel) and 7 days (right) post‐injection. Results shown are representative of mice from 3 independent experiments

To determine whether transplantation of BM‐MSCs is able to prolong the life span of *Ercc1* ^−/−^ mice, ~10^6^ BM‐MSCs from young mice were injected IP into *Ercc1* ^−/−^ mice, and their body weight and life span monitored. Treatment with MSCs cultured at 3% O_2_ had only a marginal effect on overall life span of the *Ercc1* ^−/−^ mice (data not shown; see Figure 2a). Preliminary analysis of the cells following injection suggested that the cells were not surviving post‐transplantation (data not shown), therefore, young and old MSCs were subjected briefly to oxidative stress (20% O_2_ for 48 hrs) to improve their survival following transplantation. Gene ontology analysis of the transcriptomes of young oxidatively stressed MSCs confirmed a significant enrichment of pathways regulating cell survival (Figure 2b). Injection of oxidatively stressed BM‐MSCs from young mice significantly extended life span (Figure 2a), while IP injection of their aged counterparts had a minimal effect. To document cellular survival and localization following transplantation, MSCs modified to express a highly sensitive reporter (pRETOX‐TIGHT‐GpNLuc) were utilized. At 4 days post‐injection, there was no evidence of migration and engraftment of MSCs outside the site of injection in the peritoneal cavity (Figure 2c, left), suggesting that a paracrine mechanism is responsible for the observed life‐extending effect. Furthermore, after one week post‐transplantation, only the oxidatively stressed BM‐MSCs from young mice perdured in the peritoneal cavity, suggesting that brief exposure to oxidative stress improved cell survival (Figure 2c, right). Interestingly, the mRNAs important for cellular survival differentially regulated between unstressed and stressed (20% O_2_) MSCs (Figure S2a) were distinct from those identified as differentially regulated between young and old MSCs (Figure S2b).

### 2.3 Extracellular vesicles in CM from young MSCs can reduce cellular senescence and improve stem cell function

Numerous studies have documented that the accumulation of senescent cells with age drives age‐related pathologies. Compounds that specifically ablate senescent cells (senolytics) or suppress the senescent phenotype (senomorphics) can extend health span in mouse models of accelerated and natural aging (Niedernhofer & Robbins, 2018). To identify senotherapeutic agents, we previously developed a screen for compounds able to suppress senescence in BM‐MSCs and murine embryonic fibroblasts (MEFs) specifically (Fuhrmann‐Stroissnigg, Fuhrmann‐Stroissnigg, et al., 2017; Fuhrmann‐Stroissnigg et al., 2019). To determine if factors released by young MSCs have senotherapeutic activity, conditioned media (CM) from young and old MSCs were tested for activity on oxidative stress‐induced senescent *Ercc1* ^−/−^ murine embryonic fibroblasts (MEFs) and BM‐MSCs from aged mice.

**FIGURE 3:**
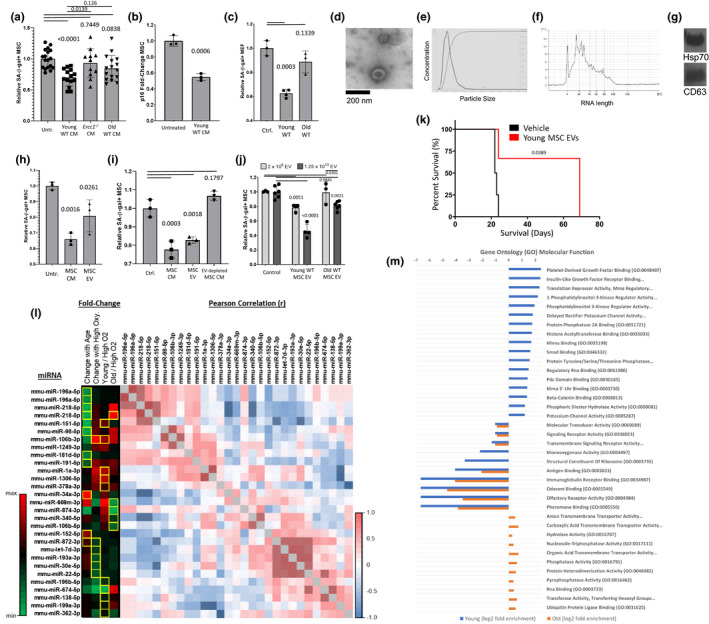
Extracellular vesicles isolated from CM of young BM‐MSCs reduce cellular senescence and improve stem cell function *in vitro* and extend life span of progeroid Ercc1‐deficient mice. (a) Senescence was induced in cultures of BM‐MSCs from aged mice by passaging, and senescent cells were treated with conditioned media (CM) from the indicated BM‐MSC cultures. Bar graph shows normalized mean ±SD from 3 independent experiments. Significance was determined by one‐way ANOVA (*p* = 0.0002, *F*(3,55) = 7.630) with Tukey's multiple comparisons test; *p* values for specific comparisons shown in figure. (b) RT‐PCR analysis of p16^INK4a^ expression in senescent young MSCs treated with conditioned media from functional non‐senescent MSCs; gene expression was normalized to GAPDH. Significance was determined by two‐tailed parametric unpaired t test (*p* = 0.0006, *t*(4) = 10.03); *p* value shown in figure. (c) Senescent cultures of ERCC1‐deficient MEFs induced by passage and oxidative stress at 20% O_2_ were treated with conditioned media from the indicated BM‐MSC cultures. Bar graph shows normalized mean ±SD from 3 independent experiments. ** *p* < 0.01. Significance was determined by one‐way ANOVA (*p* = 0.0003, *F*(2,7) = 32.69) with Tukey's multiple comparisons test; *p* values for specific comparisons shown in figure. (d) Electron micrograph of extracellular vesicles (EVs) in the conditioned media (CM) from BM‐MSCs from young mice. (e) Size distribution analysis of extracellular vesicles in the conditioned media from BM‐MSCs from young mice by NTA. (f) Analysis of the size of RNA content of EVs in the conditioned media from BM‐MSCs from young mice using an Agilent Bioanalyzer 2100. (g) Immunoblot analysis of Hsp70 and CD63 in EVs from conditioned media from BM‐MSCs from young mice. (h) Senescent MSCs from old mice were treated with CM or EVs isolated from the CM from BM‐MSCs from young mice. Bar graph shows normalized mean ±SD from 3 independent experiments. Significance was determined by one‐way ANOVA (*p* = 0.002, *F*(2,6) = 20.78) with Tukey's multiple comparisons test; *p* values for specific comparisons shown in figure. (i) Senescent BM‐MSCs from old mice were treated with CM, EVs isolated from the CM, or CM depleted of EVs from BM‐MSCs from young mice. Bar graph shows normalized mean ±SD from 3 independent experiments. Significance was determined by one‐way ANOVA (*p* < 0.0001, *F*(3,8) = 43.66) with Tukey's multiple comparisons test and t test; *p* values for specific comparisons shown in figure. (j) Senescent BM‐MSCs from old mice were treated with 2 x 10^9^ EV particles (light bar) or 1.25 x10^10^ EV particles (dark bar) from the conditioned from BM‐MSCs from young or old mice. Bar graph shows normalized mean ±SD from 3 independent experiments. Significance was determined by two‐way ANOVA with Tukey's multiple comparisons test; *p* values for specific comparisons shown in figure. Detailed ANOVA and multiple comparison results available in source data file. (k) IP injection of young MSC‐derived extracellular vesicles extends life span of *Ercc1* ^−/−^ mice. Significance of survival curves was computed by log‐rank (Mantel‐Cox) test (*p* = 0.0389, χ^2^ = 4.265, df = 1); *p* value shown in figure. (l) miRNAs identified from RNA‐seq of EVs from young and old, low and high O_2_ cultured MSCs reveals miRNAs that are co‐regulated and enriched in young or old MSC EVs, and during oxidative stress. Significant (*p* < 0.05) fold‐change miRNAs are highlighted in yellow. Matrix of Pearson correlation of miRNAs expressed in all conditions reveals groups of miRNAs that are significantly (hatched cells 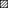) correlated. (m) Gene ontology (GO; molecular function) of miRNA targetomes for miRNAs enriched in young and old MSC EVs; axis is log_2_ of fold enrichment and all categories are significant (*p* < 0.05) after false‐discovery rate (FDR) correction. Raw miRNA read counts, pairwise Pearson *r* and *p* values, and confidence intervals available in source data file

**FIGURE 4:**
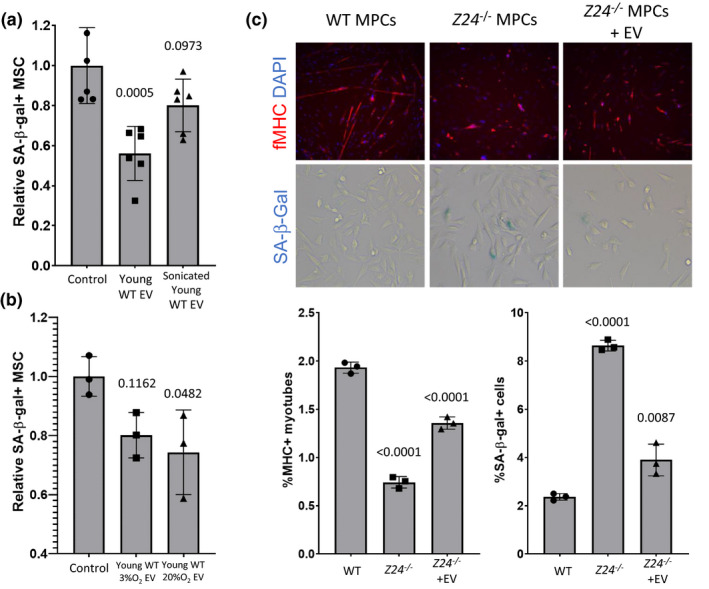
Structurally intact extracellular vesicles from young MSCs suppress senescence and restore progenitor cell function. (a) >50% senescent MSC cultures were treated with EVs from young MSCs and sonicated EVs derived from young MSC. Bar graph shows normalized mean ±SD from 3 independent experiments. Significance was determined by one‐way ANOVA (*p* = 0.0007, *F*(2,15) = 12.19) with Tukey's multiple comparisons test; *p* values for specific comparisons shown in figure. (b) >50% senescent MSC cultures were treated with extracellular vesicles from young MSCs and with extracellular vesicles from MSC briefly stimulated with oxidative stress. Bar graph shows normalized mean ±SD from 3 independent experiments. Significance was determined by one‐way ANOVA (*p* = 0.0475, *F*(2,6) = 5.282) with Tukey's multiple comparisons test; *p* values for specific comparisons shown in figure. (c) The increase in senescence in muscle progenitor cells from the *Zmpste24* ^−/−^ murine model of Hutchinson Guildford progeria syndrome was reduced by treatment with EVs derived from young WT muscle‐derived progenitor cells (MDSPCs). Fluorescent microscopy images of WT and untreated and treated *Zmpste24* ^−/−^ MDSPCs (upper left panel) show levels of fibrillar myosin heavy chain (fMHC, red) and nuclear DAPI staining (blue); levels of SA‐β‐gal staining for each condition are shown (blue X‐gal, lower left panel). Graphs expressing quantitation of percent myotube formation and SA‐β‐gal staining are shown (upper and lower right panel, respectively). Significance of quantitative data was determined by one‐way ANOVA: ANOVA**_%MHC+_** (*p* < 0.0001, *F*(2,6) = 293.2) and ANOVA**_%SA‐_**β**_‐gal_** (*p* < 0.0001, *F*(2,6) = 193.9) with Tukey's multiple comparisons test; *p* values for specific comparisons shown in figure

The addition of CM from young BM‐MSCs, but not from old or *Ercc1* ^−/−^ MSCs, decreased the percentage of SA‐β‐gal positive senescent BM‐MSCs (Figure 3a) and MEFs (Figure 3c), and reduced the expression of *p16^INK4a^*, a marker of senescence (Figure 3b). However, there was no difference in the extent of reduction of senescence between CM from young MSCs grown at 3% O_2_ versus MSCs subjected briefly to oxidative stress (data not shown; see Figure 4b). These results are consistent with young stem cells secreting factor(s) able to suppress the senescent phenotype and thus functioning as a senomorphic(s).

To determine whether EVs were among the factor(s) secreted by young, but not old MSCs that confer rescue of senescence in MEFs and BM‐MSCs, we purified the EV‐enriched high molecular weight (HMW) fraction of the conditioned media by ultracentrifugation at 100,000× g. This HMW fraction is comprised of an enriched population of EVs with a size of approximately 100 nm as demonstrated by electron microscopy (EM) (Figure 3d) and nanoparticle tracking analysis (NTA, Nanosight) (Figure 3e). The purified EVs were enriched in small RNA species, ranging from 4 to 100 bases in length and, in particular, species of approximately 20 nucleotides in length (Figure 3f). In addition, the purified EVs contained the EV markers Hsp70 and CD63, which are also markers of exosomes (Figure 3g). The addition of the EV‐enriched fraction to unconditioned media was able to reduce senescence of MSCs as efficiently as the CM (Figure 3h and 3i). Conversely, CM from young BM‐MSCs depleted of extracellular vesicles was unable to reduce the percentage of senescent aged BM‐MSCs (Figure 3i). In addition, the ability of extracellular vesicles to reduce senescence was dose dependent, with 1.25 × 10^10^ EV particles from young MSCs having a greater suppressive effect than 2 × 10^9^ EV particles (Figure 3j) in comparison with old MSC EVs. Further, EVs purified from BM‐MSC CM were able to affect life span similarly to the MSCs, as 3 × 10^9^ vesicles injected IP into *Ercc1* ^−/−^ mice at 7 and 10 days were able to extend the life span of *Ercc1* ^−/−^ mice by more than twofold (Figure 3k), suggesting that the ability of young MSCs to extend life span and health span is partly through the release of extracellular vesicles.

To further characterize the nucleic acid cargo of the MSC EVs, EVs from young and old, oxidatively stressed and unstressed MSCs were subjected to RNA‐seq analysis. This analysis identified clusters of co‐regulated miRNAs that were enriched in either young or old MSC EVs, and at either high or low oxygen conditions. This correlation analysis identified groups of microRNAs (miRNAs) whose expression was positively or negatively correlated (Figure 3l). A comparative gene ontology analysis of the targetomes of young and old MSC EV miRNA (blue and orange, respectively) profiles revealed distinct target sets (Figure 3m). Closer inspection of the top fold‐enriched GO molecular function categories reveals that young MSC EV miRNAs strongly converge upon targets participating in the PDGF‐PI3 K‐IIGF (platelet‐derived growth factor; phosphoinositide 3‐kinase; insulin and insulin‐like growth factors) signaling axis (Figure S3a).

To confirm a role for EVs in conferring the effect on senescence, the effect of disrupting the intact EV particles was examined. As shown (Figure 4a), sonication of 1.25 × 10^10^ EVs from young MSCs reduced their senomorphic activity in comparison with the same number of unsonicated EVs, suggesting that intact vesicles are important for conferring at least part of the effect on stem cells. Further, EVs from young MSCs grown at 3% O_2_ or briefly exposed to oxidative stress (20% O_2_) had similar senomorphic activity when applied to aged MSC cultures (Figure 4b). We previously demonstrated that CM from young, but not old MDSPCs can rescue the ability of *Ercc1* ^−/−^ MDSPCs to proliferate and to differentiate into myoblasts. EVs isolated from the conditioned media of young MDSPCs were able to reduce senescence and improve differentiation of MDSPCs isolated from the *Zmpste24* ^−/−^ mouse model of Hutchinson‐Gilford progeria syndrome (HGPS), which also rapidly undergo senescence in culture (Figure 4c).

### 2.4 EVs from hESC‐MSCs have senomorphic activity in vitro and regulate expression of genes implicated in aging

**FIGURE 5:**
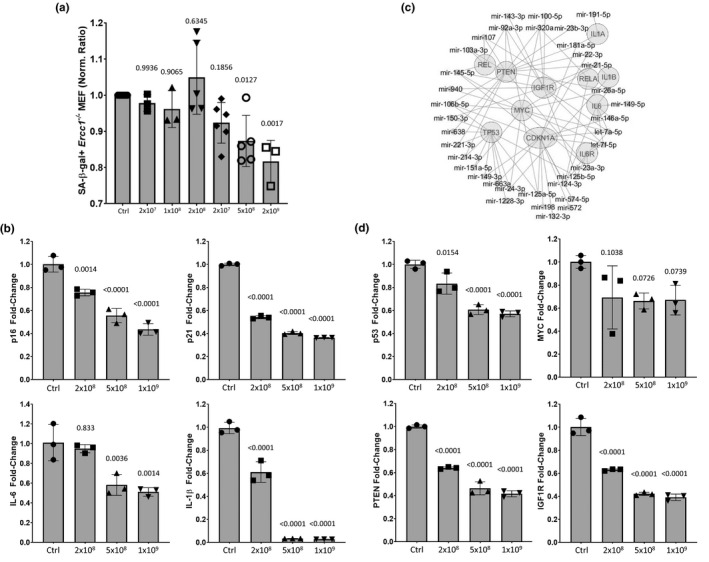
Extracellular vesicles from human embryonic stem cell‐derived MSCs (AC83 EVs) reduce senescence *in vitro*. (a) Increasing particle numbers of AC83 EVs were added to senescent cultures of MEFs and percent senescent cells determined by C_12_FDG staining 72 hours post‐treatment. Significance was determined by one‐way ANOVA (*p* < 0.0003, *F*(6,24) = 6.79) with Dunnett's multiple comparisons tests; *p* values for specific comparisons shown in figure. (b) RT‐PCR analysis of senescent markers p16^INK4a^ and p21^Cip1^ and SASP factors IL‐6 and IL‐1β measured in senescent MEFs 72 hours post‐treatment with the indicated number of AC83 EVs. Significance was determined by one‐way ANOVA: ANOVA**_p16_** (*p* < 0.0001, *F*(3,8) = 63.34), ANOVA**_p21_** (*p* < 0.0001, *F*(3,8) = 2277), ANOVA**_IL‐6_** (*p* = 0.001, *F*(3,8) = 15.89) and ANOVA**_IL‐1_**β (*p* < 0.0001, *F*(3,8) = 254.3) with Dunnett's multiple comparisons test; *p* values for specific comparisons shown in figure. (c) Radial miRNA‐mRNA interactome demonstrates the most highly enriched miRNAs in AC83 EVs and their predicted targets. miRNAs are highly convergent upon the most centrally located targets. (d) RT‐PCR analysis of p53, PTEN, IGF‐1R, and MYC measured in senescent MEFs 24 hours post‐treatment with the indicated number of AC83 EVs. Significance was determined by one‐way ANOVA: ANOVA**_p53_** (*p* < 0.0001, *F*(3,8) = 39.85), ANOVA**_MYC_** (*p* < 0.0827, *F*(3,8) = 3.217), ANOVA**_PTEN_** (*p* < 0.0001, *F*(3,8) = 203) and ANOVA**_IGF1R_** (*p* < 0.0001, *F*(3,8) = 145.1) with Tukey's multiple comparisons test; *p* values for specific comparisons shown in figure. Data shown are representative of four independent experiments with similar results

Expansion of murine BM‐MSC in culture results in higher‐passage cells that secrete EVs with diminishing senomorphic potency, limiting the quantity of functional EVs that can be purified for multiple mouse treatments for analysis of effects on senescence and health span. Thus, CM and EVs from multiple sources of mouse and human stem cells were screened in the MEF senescence assay (data not shown). Interestingly, EVs derived from human MSCs generated by differentiation of ES cells (hESC‐MSC EVs, batch identifier “AC83”) were consistently effective at reducing the percentage of SA‐ß‐gal+senescent MEFs (Figure 5a) and IMR‐90 fibroblasts (Figure S4) in culture. To further document the suppression of senescence by AC83 EVs, the effects on expression of the senescence markers p16^INK4a^ and p21^Cip1^ and the SASP factors, IL‐6, and IL‐1β were examined by RT‐PCR. As shown (Figure 5b), treatment with AC83 EVs reduced the expression of p16^INK4a^, p21^Cip1^, and SASP factors, with almost complete suppression of IL‐1β expression.

Analysis of the top miRNAs enriched in AC83 EVs (Chen et al., 2010) identified a convergence of miRNA targets including p21^Cip−1^, PTEN, MYC, p53, IGF‐1R, IL‐6, IL‐6R, REL, IL‐1β, IL‐1β, and REL‐A (Figure 5c). Furthermore, AC83 miRNAs (Figure S3b) and MSC EV miRNAs share an overrepresentation of targets in the PDGF/PI3 K/IIGF signaling axis. RT‐PCR analysis of senescent MEFs treated with AC83 EVs showed a dose‐dependent reduction in the expression of p53, PTEN, MYC, and IGF‐1R (Figure 5d). These results demonstrate that hESC‐MSC‐derived EVs were able to suppress not only biomarkers of senescence, but also other factors such as MYC and IGF‐1R that have been implicated in aging.

### 2.5 EVs from human embryonic stem cell‐derived MSCs suppress senescence in vivo and extend health span

**FIGURE 6:**
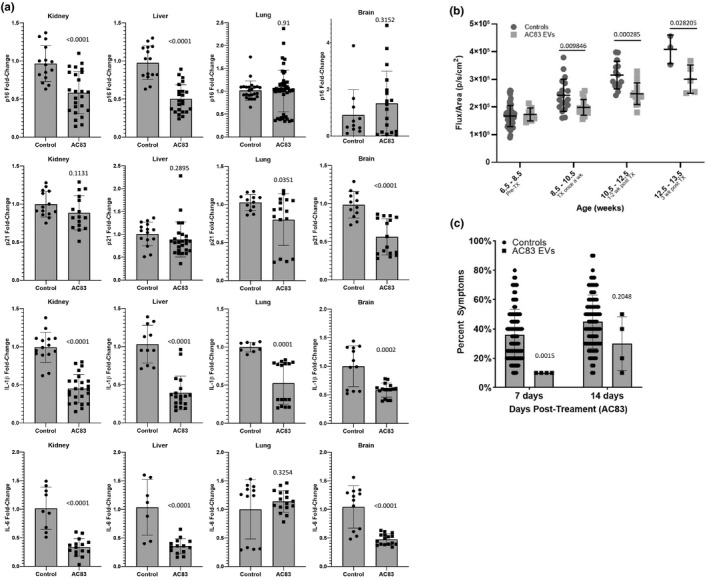
Extracellular vesicles from human embryonic stem cell‐derived MSCs (AC83 EVs) reduce markers of senescence *in vivo*, and suppress senescence and improve healthspan *in vivo*. (a) Naturally aged wild‐type mice were IP injected twice with 10^9^ AC83 EVs. Analysis of gene expression in kidney, liver, lung, and brain by RT‐PCR revealed decreases in expression of p16^INK4a^ and p21^Cip1^, IL‐6, and IL‐1β. Significance was determined by two‐tailed parametric unpaired t tests: t test**_p16_kidney_** (*p* < 0.0001, t(38) = 4.425), t test**_p16_liver_** (*p* < 0.0001, t(36) = 7.105), t test**_p16_lung_** (*p* = 0.91, t(69) = 0.1135), t test**_p16_brain_** (*p* = 0.1062, t(5) = 1.968), t test**_p21_kidney_** (*p* = 0.1331, t(28) = 1.547), t test**_p21_liver_** (*p* = 0.2895, t(38) = 1.074), t test**_p21_lung_** (*p* = 0.0351, t(26) = 2.223), t test**_p21_brain_** (*p* < 0.0001, t(26) = 5.193), t test**_IL‐1_**β**__kidney_** (*p* < 0.0001, t(37) = 8.647), t test**_IL‐1_**β**__liver_** (*p* < 0.0001, t(28) = 7.287), t test**_IL‐1_**β**__lung_** (*p* = 0.0001, t(22) = 4.732), t test**_IL‐1_**β**__brain_** (*p* < 0.0002, t(26) = 4.382), t test**_IL‐6_kidney_** (*p* < 0.0001, t(22) = 6.381), t test**_IL‐6_liver_** (*p* < 0.0001, t(19) = 4.983), t test**_IL‐6_lung_** (*p* = 0.3254, t(26) = 1.002), t test**_IL‐6_brain_** (*p* < 0.0001, t(26) = 5.920); *p* values shown in figure. Data shown are representative of independent experiments conducted on three cohorts of aged mice. (b) *Ercc1* ^−/^ *^∆^* mice expressing a p16^INK4a^‐luciferase reporter were given two IP injections of 10^9^ AC83 EVs, resulting in a relative decrease in p16^INKa^‐mediated luciferase expression compared to control animals. Significance was determined by multiple t tests; *p* values shown in figure. Detailed multiple t test results available in source data file. (c) *Ercc1* ^−/^ *^∆^* mice given two IP injections of 10^9^ AC83 EVs and control animals were scored for measures of health span, at 7‐ and 14‐days post‐treatment, including ataxia, body condition, dystonia, gait disorders, kyphosis and tremor. Progeroid *Ercc1* ^−/^ *^∆^* mice treated with AC83 EVs demonstrated consistent improvement in health span compared to control animals. Significance was determined by Šidák's multiple comparisons test; adjusted *p* values shown in figure. Detailed multiple comparisons test results available in source data file. Data from (b) were generated from 8, 16, 16, and 5 mice per age group, respectively. Data from (c) were generated from 4 mice per group

To evaluate the penetrance of the senotherapeutic activity of AC83 EVs *in vivo*, 2‐year‐old wild‐type C57/Bl6 mice (108 weeks) were treated with two IP injections of 5 × 10^9^ AC83 EVs, at days 0 and 7, and then sacrificed at day 10 (Figure S5a). RT‐PCR revealed significant decreases in expression of the senescence markers p16^INK4a^ and p21^Cip1^, and the SASP factors, IL‐6, and IL‐1β in multiple tissues of treated mice (Figure 6a). Interestingly, and in concordance with *in vitro* data, AC83 EVs were also capable of suppressing expression of p53, PTEN, MYC, and IGF‐1R in brain, kidney, and lung of treated naturally aged wild‐type mice (Figure S5b), suggesting a similar therapeutic mechanism of action *in vitro* and in different tissues *in vivo*.

To further document the suppression of senescence *in vivo*, AC83 EVs were tested in *Ercc1* ^−/∆^ mice carrying a p16^INK4a^‐luciferase reporter (*p16^luc^* ^/+^; *Ercc1* ^−/∆^) (Robinson et al., 2018). Previous experiments have established that p16^INK4a^‐luciferase expression increases with accelerated aging in the same tissues as naturally aged mice (Burd et al., 2013). Two IP injections of 10^9^ AC83 EVs into *Ercc1* ^−/∆^ mice carrying a p16^INKa^‐luciferase senescence reporter resulted in a significant reduction of p16^INKa^‐mediated luciferase expression (Figure 6b), consistent with the observation of reduced *p16^INK4a^* transcription in naturally aged wild‐type mice.

Progeroid *Ercc1* ^−/∆^ mice, which rapidly accrue a large senescent cell burden in multiple tissues similar to old WT mice and develop accelerated age‐related pathologies, are an excellent model for evaluating health span. To document the effects of AC83 EVs on health span, two IP injections of 10^9^ AC83 EVs were administered (Figure S6a) and animals monitored for changes in multiple parameters of health. Progeroid *Ercc1* ^−/∆^ mice treated with AC83 EVs scored consistent with a significant improvement in aggregate (percent) symptoms compared to control mice (Figure 6c) and demonstrated a significant reduction in age‐related weight loss (Figure S6). Taken together, these *in vivo* results suggest that adult stem cell EVs can suppress markers of senescence and extend health span, similarly to the results observed with senotherapeutic compounds.

## 3 DISCUSSION

Numerous studies suggest that the number and function of diverse somatic stem cell populations decline with age. However, there is limited evidence for loss of stem cell function as a primary driver of age‐related pathology and lifespan abbreviation, contrasted with it being merely a secondary consequence of aging. Our previous observations suggested that young MDSPCs were capable of healthspan and lifespan extension via the secretion of stimulatory factor(s) that conferred regeneration, a therapeutic activity that is lost with age. To extend these to other adult stem cells types, we examined the effect of age on MSC function and the ability of young, functional MSCs to extend life span. Here, we demonstrate that BM‐MSCs from naturally aged and progeroid *Ercc1* ^−/−^ mice have a reduced proliferative capacity in culture, with a corresponding increase in markers of senescence including SA‐ß‐gal, with similar results observed for MDSPCs (Figure 4c). Similar to MDSPCs, IP injection of young BM‐MSCs extended life span in *Ercc1* ^−/−^ mice. Survival of IP‐injected young MSCs was contingent upon brief oxidative stress, likely reflecting the induction of a stress response leading to the upregulation anti‐apoptotic survival pathways (Abramowicz et al., 2019).

As previously shown for CM from MDSPCs, CM from young, but not old MSCs reduced expression of markers of senescence in aged MSCs and senescent MEFs, including SA‐ß‐gal, p16^INK4a^ expression. Also, the mode of action was the suppression of the senescent phenotype (senomorphism) rather than the specific ablation of senescent cells (senolysis). The senomorphic activity of young MSC CM co‐purified with EVs isolated from the MSC CM. The CM from old MSCs lacked this senomorphic potency, suggesting that the age‐related dysfunction of MSCs includes a decline in the activity and abundance of EVs with age (Chen et al., 2013). Similarly, EVs from MDSPCs also reduced senescence and improved myogenesis in MDSPCs isolated from the *Zmpste24* ^−/−^ mouse model of HGPS. EVs are comprised of both microvesicles and nanovesicles, or exosomes, which are characterized predominantly by their size. EM and NTA analysis of the EVs from BM‐MSCs revealed a size distribution of around 100 nm, consistent with exosomes, and the EVs also contained exosome marker proteins and were enriched for small RNAs.

Two IP injections of EVs extended the life span of *Ercc1* ^−/−^ mice similarly to injection of the parental young MSCs. Importantly, EVs derived from hESC‐MSCs reduced expression of the p16^INK4^‐luciferase transgene reporter, and senescence and SASP markers significantly, documenting that they have *in vivo* senomorphic activity. The results suggest that the hES‐MSC (AC83) EVs reduce p16^INK4a^‐luciferase expression in the *Ercc1* ^−/^ *^∆^* mice more efficiently than treatment with known senomorphics, which require constant dosing, and senolytics able to induce apoptosis of senescence cells with intermittent treatment. In particular, the EVs were at least as effective at reducing luciferase expression in the *p16^luc^* ^/+^; *Ercc1* ^−/^ *^∆^* mice as the combination of dasatinib and quercetin (D + Q) or the Bcl‐2 inhibitor Navitoclax (data not shown), both of which have been reported to have beneficial effects on health span and, at least for D + Q, life span. Thus, stem cell EVs could extend healthspan via suppression of senescence. However, the fact that the hES‐MSC EVs suppressed expression of p53, PTEN, IGF‐1R, and MYC suggests that other mechanisms important for aging may be involved (Hofmann et al., 2015; Tazearslan et al., 2011; Wu & Prives, 2018). Insulin signaling has been demonstrated to regulate aging and longevity across species and the prolonged activation of this signaling axis is implicated in p53‐mediated cellular senescence (Tran et al., 2014). We propose that the ability of stem cell EVs to extend life span and health span is through modulation of senescence and, in particular, SASP that drives secondary senescence. However, it remains to be determined to what extent the MSC EVs may have contributed to the improvement in health span and life span of treated animals via their documented regenerative effects upon oxidative stress reduction, anti‐inflammation, survival kinase signaling (Arslan et al., 2013), restoration of immune homeostasis (Zhang, Yeo, et al., 2018), cellular proliferation, enhancement of extracellular matrix and immune function (Zhang, Chuah, et al., 2018), and stem cell regenerative capacity (Chew et al., 2019; Tan et al., 2014; Toh et al., 2018; Zhang et al., 2016; Zhang, Chuah, et al., 2018). The mechanism by which EVs suppress senescence in culture and *in vivo* is unclear, but likely involves the convergent activity of specific miRNAs. These observations do not preclude the involvement of lipids, proteins, and metabolites in the MSC exosomes (Pathan et al., 2019; Toh et al., 2018) as contributors to their senotherapeutic effect, and the magnitude and therapeutic mechanism of EVs may vary depending on the progenitor cell source, as well as their target tissues. For example, preadipocyte‐derived EVs carrying eNAMPT have been demonstrated to increase NAD+levels (Yoshida et al., 2019).

Previously, we have used heterochronic parabiosis to demonstrate that circulating factors increase or suppress senescence (Yousefzadeh et al., 2020), results consistent with a role for circulating EVs in regulating senescence systemically. Overall, our results demonstrate a role for EVs released by functional stem cells in modulating senescence and possibly other pathways related to longevity and aging. Stem cell‐derived EVs allow for the tight regulation of the duration and dosage of treatment and their use precludes the risks of tumor development and donor cell rejection. Given that human ES or iPS cells can be expanded and differentiated in bioreactors to generate high yields of progenitor cell‐derived EVs, the therapeutic potential for adult stem cell EVs in the treatment of age‐related pathologies is promising.

## 4 EXPERIMENTAL PROCEDURES

### 4.1 MSC tracking

Injected MSC was tracked *in vivo* using luciferase activity. Luc‐GFP plasmid transfected MSC were injected IP and whole‐mouse images taken using the IVIS Xenogen imager system at the indicated time points.

### 4.2 MSC EV isolation

EVs were isolated by ultracentrifugation. Briefly, the supernatant (conditioned media) of MSC cultured during indicated period was harvested and the cell debris removed by centrifugation of the samples to 7000× g for 20 min. Supernatants were then pre‐cleared of microvesicles by centrifugation at 16,500× g for 2 hrs at 4ºC, after which the EVs were isolated by ultracentrifugation at 100,000× g for 2 hrs at 4ºC, washed with PBS once, and recentrifuged at 100,000× g for an additional 2 hrs at 4ºC. EV concentrations and homogeneity were quantified using Nanosight technology and electron microscopy.

### 4.3 Human embryonic stem cell‐derived MSC EV isolation

The hESC‐MSC EVs were prepared as previously described (Lai et al., 2016). Briefly, immortalized hES‐ MSCs were grown in a chemically defined medium for 3 days and CM was harvested and pre‐cleared with a 0.22 µm syringe filter. The CM was concentrated 100× for exosomes by tangential flow filtration (Sartorius; MWCO 100 kDa) and stored in −20°C freezer until use. The EVs were assayed for protein concentration using Coomassie Plus (Bradford) Assay (Thermo Fisher Scientific) per manufacturer's instruction and characterized for particle size distribution and concentration by Zetaview (Particle Metrix) according to the manufacturer's protocol. Follow‐up experiments from subsequent batches of hESC‐MSC EVs (posterior to batch AC83) yielded identical results.

### 4.4 MSC isolation

MSC was obtained from *Ercc1* ^−/−^ and WT mice bone marrow and cultured in high glucose DMEM supplemented with 15% FBS, 2 mM glutamine, 100 U/ml penicillin, and 0.1 mg/ml streptomycin (all from Sigma‐Aldrich, St Louis, MO, USA). The MSC were cultured in low oxygen conditions (3% O_2_) to avoid oxidative damage. The bone marrow (BM) cells were flushed out from the long bones using a syringe after euthanization. Extracted BM cells were washed by centrifugation and seeded at 2.5 ×10^5^ cells/cm^2^ (passage “0”). Non‐adherent cells were discarded by changing the media at 16 h and the culture enriched for MSCs by culturing cells for 3 passages. The generated MSCs display CD105+, CD106+, CD73+, Sca‐1+, CD34‐ CD45‐, CD31‐ phenotype, fibroblast‐like morphology, and multilineage differentiation capacity. Adipogenic medium includes 1 mM dexamethasone, 5 μg/ml insulin, and 200 mM indomethacin (Sigma). For adipogenic quantification, the cell monolayer was fixed (10% formalin) and stained with 0.2% Oil Red O (Sigma). The dye bound to lipids is extracted and quantified by reading OD_520 nm_ and the values normalized to DNA content. Osteogenic medium contained 50 mM ascorbic acid, 10 mM b‐glycerophosphate, and 0.1 mM dexamethasone (Sigma). For osteogenic visualization, cells were fixed (70% ethanol) and stained in Alizarin Red S (2%) for 10 minutes. Stained cells were washed twice (dH_2_O).

### 4.5 Clonogenic capacity assay

The clonogenic capacity of BM cells was measured at passage 0. Six days after seeding passage 0 cells, the cells were fixed (PFA 2%) and stained with Crystal Violet 0.05% (Sigma‐Aldrich) for 30 min and clones counted manually.

### 4.6 Senescence assay

Cell senescence was assayed by measurement of senescence specific β‐galactosidase activity, using both conventional X‐gal and fluorescent C_12_FDG as SA‐β‐galactosidase substrates. For the chromogenic senescence assay, cells were seeded at 5000 cells/cm^2^ and treated for 48 hrs with the desired treatment. The cells were fixed (PFA 2%) for 5 min and stained overnight with X‐gal (2 mg/ml) staining buffer pH 6.0 (Teknova) at 37ºC. Nuclei were labeled with DAPI 1 µg/ml (Life Technologies) and β‐gal positive cells were counted manually. The C_12_FDG‐based senescence assay was performed as described (Fuhrmann‐Stroissnigg, Fuhrmann‐Stroissnigg, et al., 2017; Fuhrmann‐Stroissnigg et al., 2019).

### 4.7 Treatment of mice with EVs

Experimental procedures involving animals were performed in strict observance of the regulatory standards outlined in the U.S. Department of Health and Human Services Guide for the Care and Use of Laboratory Animals and with the approval of the Institutional Animal Care and Use Committees at the University of Minnesota and Scripps Florida. Wild‐type mice were administered 5 × 10^9^ AC83 EVs by IP injection, twice as indicated (Figure S6a). *Ercc1* ^−/−^ mice were given two IP injections of 10^9^ EVs at indicated ages. For health span, *Ercc1* ^−/Δ^ mice were weighed twice a week and monitored for the onset of age‐related symptoms, including dystonia, trembling, ataxia, priapism and urinary incontinence, hind‐limb muscle wasting, lethargy, and kyphosis.

### 4.8 IVIS in vivo imaging detection of luciferase activity

After treatment as described (Figure S6a), isoflurane‐anesthetized *p16^luc^* ^/+^; *Ercc1* ^−/Δ^ mice were injected intraperitoneally with D‐luciferin (Caliper Life Sciences; 15 mg/mL in PBS) and imaged in an IVIS Xenogen (Caliper Life Sciences) as previously described (Niedernhofer & Robbins, 2018). Briefly, animals were anesthetized starting at 3%‐5% Isoflurane and decreasing to 1–3% for maintenance sedation. 10 µL/g‐BW D‐luciferin solution was administered per mouse subcutaneously. After 10 min, IVIS began collecting measurements every 2 min until a maximum of 20 min (6 acquistions). Raw data for each mouse were processed using a Microsoft Excel macro that divides total flux per animal by region‐of‐interest (ROI) area and subtracts background flux/area from the animal flux value.

### 4.9 Relative gene expression by RT‐PCR

Snap‐frozen tissues from mice were homogenized in TRIzol (Invitrogen) in a Lysing matrix D tube (MP Bio) with the FastPrep‐24/5^G^ homogenizer. RNA was extracted according to manufacturer instruction (Invitrogen). Two micrograms of RNA were used to generate cDNA using the High Capacity RNA‐to‐cDNA kit (Applied Biosystems) and standard RT‐PCR reactions were performed using PowerUp SYBR Green master mix using a minimum 20 ng RNA equivalents per reaction. Forward and reverse oligonucleotide primers were used at a final concentration of 500 nM per reaction. A panel of 13 housekeeping genes (GAPDH, ACTB, B2 M, HMBS, HPRT, RpLp0, TBP, GUSB, PPIA, OAZ1, NONO, TFRC, and EEF2) was used for normalization, and the most stable pair of housekeeping genes was determined for each tissue as described (Hellemans & Vandesompele, 2014). Housekeeping gene primer sequences were previously described (Eissa et al., 2016). For the senescence markers p16^INK4a^, p21^Cip−1^, IL‐6, and IL‐1β, a panel of published and validated primers for each gene was used to reliably and reproducibly detect changes in gene expression; the primer sequences were previously described (source data file Table S1 for sequences and references). Sequences of additional primers used are as follows: PTEN forward 5'‐TGGATTCGACTTAGACTTGACCT‐3’, PTEN reverse 5'‐GCGGTGTCATAATGTCTCTCAG‐3’, MYC forward 5'‐ATGCCCCTCAACGTGAACTTC‐3’, MYC reverse 5'‐CGCAACATAGGATGGAGAGCA‐3’, p53 forward 5'‐GCGTAAACGCTTCGAGATGTT‐3’, p53 reverse 5'‐TTTTTATGGCGGGAAGTAGACTG‐3’, IGF1R forward 5'‐CATGTGCTGGCAGTATAACCC‐3’, IGF1R reverse 5'‐TCGGGAGGCTTGTTCTCCT‐3’, p21 #4 forward 5’‐TCGCTGTCTTGCACTCTGGTGT‐3’, p21 #4 reverse 5’‐CCAATCTGCGCTTGGAGTGATAG‐3’. RT‐PCR data were analyzed using the delta‐delta Ct method (Livak & Schmittgen, 2001).

### 4.10 miRNA Interactome

The miRNAs previously identified in hESC‐MSC EVs (Chen et al., 2010) were queried for miRNA‐target interaction using the miRNet collection of validated miRNA targets (Fan & Xia, 2018). An interactome was compiled from raw miRNA‐target data using the Cytoscape bioinformatics software platform (Shannon et al., 2003).

### 4.11 Bioinformatic analysis

Normalized read counts of RNA‐seq data underwent Ingenuity Pathway Analysis (IPA; Qiagen) to determine the gene ontologies enriched in the differentially expressed sets of mRNAs and miRNAs. Scatter plots represent differential expression of miRNAs and mRNAs in young (y‐axis) versus old MSC EVs on a log2(fold‐change) scale. Heatmaps were generated for comparisons between young MSCs at 3% and 20% O_2_, and young versus old MSCs (at 3%) for a subset of genes with the functional annotation “Cell Death and Survival.” Heatmap dendrograms indicate genes with similar expression patterns.

### 4.12 Statistical methods and reproducibility

Statistical methods and replicates are discussed in figure legends accompanying each figure, and comprehensive and detailed statistical test results are provided in the source data file accompanying this publication. Briefly, results of ANOVA tests are reported as (*p *= pvalue, F(DFn,DFd) = Fstat) where DFn =degrees of freedom of numerator, DFd =degrees of freedom of denominator, and Fstat =F statistic. ANOVA tests were performed with Dunnett's and Tukey's post‐tests. Results of t tests are reported as (*p *= pvalue, t(df) = tstat) where df = degrees of freedom and tstat = t statistic. All *p* values for comparisons are shown in graphs.

## CONFLICT OF INTEREST

SKL is a founder of Paracrine Therapeutics, which develops EVs for therapeutic applications. PDR and LJN are co‐founders of NRTK Biosciences, which is developing approaches to reduce the senescent cell burden.

## **AUTHOR** **CONTRIBUTIONS**

AD and PDR conceived the project. AD, SVB, and DGP performed initial MSC culture, EV purification, and *in vitro* treatments. RCL and SKL developed, isolated, and provided hESC‐MSC EVs (AC83). MGC and CR performed the nanosight analysis of EV concentration and size whereas DBS performed the electron microscopy of EVs. ML and AL under the direction of JH performed the experiments with MDSPCs. FES, DG, and TZ performed *in vitro* analysis of hESC‐MSC EVs. FES and TZ performed RT‐PCR analysis of mouse tissues. TZ, AD, FES, LJN, and PDR planned the *in vivo* experiments. TZ, LA, LC, and SJM performed the experiments. AA provided the lentiviral vector expressing Shrimp luciferase and GFP. SJM performed statistical analysis on IVIS and healthspan data. MF and AR performed bioinformatic analysis on RNA‐seq data. AD, FES, and PDR involved in writing the manuscript. All the authors edited it.

## Supporting information

Fig S1‐6

Source Data S1

## Data Availability

The authors declare that all the data used to generate the findings within this study are furnished within the paper, in the supplementary information, or are available from the corresponding author upon request. A link to the source data file is included with this publication (https://doi.org/10.5281/zenodo.4317941).

## References

[acel13337-bib-0001] Abramowicz, A. , Abramowicz, A. , Widłak, P. , & Pietrowska, M. (2019). Different types of cellular stress affect the proteome composition of small extracellular vesicles: A mini review. Proteomes, 7, 23. 10.3390/proteomes7020023 31126168 PMC6631412

[acel13337-bib-0002] Arslan, F. , Lai, R. C. , Smeets, M. B. , Akeroyd, L. , Choo, A. , Aguor, E. N. , Timmers, L. , van Rijen, H. V. , Doevendans, P. A. , Pasterkamp, G. , Lim, S. K. , de Kleijn, D. P. (2013). Mesenchymal stem cell‐derived exosomes increase ATP levels, decrease oxidative stress and activate PI3K/Akt pathway to enhance myocardial viability and prevent adverse remodeling after myocardial ischemia/reperfusion injury. Stem Cell Research, 10(3), 301–312. 10.1016/j.scr.2013.01.002 23399448

[acel13337-bib-0003] Baker, D. J. , Childs, B. G. , Durik, M. , Wijers, M. E. , Sieben, C. J. , Zhong, J. , A. Saltness, R. , Jeganathan, K. B. , Verzosa, G. C. , Pezeshki, A. , Khazaie, K. , Miller, J. D. , van Deursen, J. M. (2016). Naturally occurring p16(INK4a)‐positive cells shorten healthy lifespan. Nature, 530, 184–189. 10.1038/nature16932 26840489 PMC4845101

[acel13337-bib-0004] Baker, D. J. , Wijshake, T. , Tchkonia, T. , LeBrasseur, N. K. , Childs, B. G. , van de Sluis, B. , Kirkland, J. L. , van Deursen, J. M. (2011). Clearance of p16INK4a‐positive senescent cells delays ageing‐associated disorders. Nature, 479, 232–236. 10.1038/nature10600 22048312 PMC3468323

[acel13337-bib-0005] Burd, C. E. , Sorrentino, J. A. , Clark, K. S. , Darr, D. B. , Krishnamurthy, J. , Deal, A. M. , Bardeesy, N. , Castrillon, D. H. , & Beach, D. H. Sharpless, N. E. (2013). Monitoring tumorigenesis and senescence in vivo with a p16(INK4a)‐luciferase model. Cell, 152, 340–351. 23332765 10.1016/j.cell.2012.12.010 PMC3718011

[acel13337-bib-0006] Chen, T. S. , Lai, R. C. , Lee, M. M. , Choo, A. B. H. , Lee, C. N. , & Lim, S. K. (2010). Mesenchymal stem cell secretes microparticles enriched in pre‐microRNAs. Nucleic Acids Research, 38(1), 215–224. 10.1093/nar/gkp857 19850715 PMC2800221

[acel13337-bib-0007] Chen, T. S. , Yeo, R. W. Y. , Arslan, F. , Yin, Y. , Tan, S. S. , Lai, R. C. , Choo, A. , Padmanabhan, J. , Lee, C. N. , de Kleijn, D. P. V. , & Tan, K. H. Lim, S. K. (2013). Efficiency of exosome production correlates inversely with the developmental maturity of MSC donor. Journal of Stem Cell Research & Therapy, 3, 145. 10.4172/2157-7633.1000145

[acel13337-bib-0008] Chew, J. R. J. , Chuah, S. J. , Teo, K. Y. W. , Zhang, S. , Lai, R. C. , Fu, J. H. , Lim, L. P. , Lim, S. K. , Toh, W. S. (2019). Mesenchymal stem cell exosomes enhance periodontal ligament cell functions and promote periodontal regeneration. Acta Biomaterialia, 15(89), 252–264. 10.1016/j.actbio.2019.03.021 30878447

[acel13337-bib-0009] Childs, B. G. , Baker, D. J. , Wijshake, T. , Conover, C. A. , Campisi, J. , & van Deursen, J. M. (2016). Senescent intimal foam cells are deleterious at all stages of atherosclerosis. Science, 354, 472–477. 10.1126/science.aaf6659 27789842 PMC5112585

[acel13337-bib-0010] Eissa, N. , Hussein, H. , Wang, H. , Rabbi, M. F. , Bernstein, C. N. , & Ghia, J. E. (2016). Stability of reference genes for messenger RNA quantification by real‐time PCR in mouse dextran sodium sulfate experimental colitis. PLoS One, 11(5), e0156289. 10.1371/journal.pone.0156289 27244258 PMC4886971

[acel13337-bib-0011] Fan, Y. & Xia, J. (2018). miRNet‐functional analysis and visual exploration of miRNA‐target interactions in a network context. Methods in Molecular Biology, 1819, 215–233. 30421406 10.1007/978-1-4939-8618-7_10

[acel13337-bib-0012] Fuhrmann‐Stroissnigg, H. , Fuhrmann‐Stroissnigg, H. , Ling, Y. Y. , Zhao, J. , McGowan, S. J. , Zhu, Y. , Brooks, R. W. , Grassi, D. , Gregg, S. Q. , Stripay, J. L. , Dorronsoro, A. , Corbo, L. , Tang, P. , Bukata, C. , Ring, N. , Giacca, M. , Li, X. , Tchkonia, T. , Kirkland, J. L. , Niedernhofer, L. J. , Robbins, P. D. (2017). Identification of HSP90 inhibitors as a novel class of senolytics. Nature Communications, 8(1), 422. 10.1038/s41467-017-00314-z PMC5583353 28871086

[acel13337-bib-0013] Fuhrmann‐Stroissnigg, H. , Ling, Y. Y. , Zhao, J. , McGowan, S. J. , Zhu, Y. I. , Brooks, R. W. , Grassi, D. , Gregg, S. Q. , Stripay, J. L. , Dorronsoro, A. , Corbo, L. , Tang, P. , Bukata, C. , Ring, N. , Giacca, M , Li, X. , Tchkonia, T. , Kirkland, J. L. , Niedernhofer, L. J. , Robbins, P. D. (2017). Identification of HSP90 inhibitors as a novel class of senolytics. Nature Communications, 8, 422. 10.1038/s41467-017-00314-z PMC5583353 28871086

[acel13337-bib-0014] Fuhrmann‐Stroissnigg, H. , Santiago, F. E. , Grassi, D. , Ling, Y. , Niedernhofer, L. J. , & Robbins, P. D. (2019). SA‐β‐galactosidase‐based screening assay for the identification of senotherapeutic drugs. The Journal of Visualized Experiments, 148(Jun 28), 10.3791/58133. 31305507

[acel13337-bib-0015] Hellemans, J. & Vandesompele, J. (2014). Selection of reliable reference genes for RT‐qPCR analysis. Methods in Molecular Biology, 2014, 19–26. 10.1007/978-1-4939-0733-5_3 24740218

[acel13337-bib-0016] Hofmann, J. W. , Zhao, X. , De Cecco, M. , Peterson, A. L. , Pagliaroli, L. , Manivannan, J. , Hubbard, G. B , Ikeno, Y. , Zhang, Y. , Feng, B. , Li, X. , Serre, T. , Qi, W. , Van Remmen, H. , Miller, R. A. , Bath, K. G. , de Cabo, R. , Xu, H. , Neretti, N. , Sedivy, J. M. (2015). Reduced expression of MYC increases longevity and enhances healthspan. Cell, 160, 477–488. 10.1016/j.cell.2014.12.016 25619689 PMC4624921

[acel13337-bib-0017] Jeon, O. H. , Kim, C. , Laberge, R. M. , Demaria, M. , Rathod, S. , Vasserot, A. P. , Chung, J. W. , Kim, D. H. , Poon, Y. , David, N. , David, N. , Baker, D. J. , van Deursen, J. M. , Campisi, J. , & Elisseeff, J. H. (2017). Local clearance of senescent cells attenuates the development of post‐traumatic osteoarthritis and creates a pro‐regenerative environment. Nature Medicine, 23, 775–781. 10.1038/nm.4324 PMC5785239 28436958

[acel13337-bib-0018] Kirkland, J. L. & Tchkonia, T. (2015). Clinical strategies and animal models for developing senolytic agents. Experimental Gerontology, 68, 19–25. 10.1016/j.exger.2014.10.012 25446976 PMC4412760

[acel13337-bib-0019] Kirkland, J. L. & Tchkonia, T. (2017). Cellular senescence: A translational perspective. Ebiomedicine, 21, 21–28. 10.1016/j.ebiom.2017.04.013 28416161 PMC5514381

[acel13337-bib-0020] Kirkland, J. L. , Tchkonia, T. , Zhu, Y. , Niedernhofer, L. J. , & Robbins, P. D. (2017). The clinical potential of senolytic drugs. Journal of the American Geriatrics Society, 65, 2297–2301. 10.1111/jgs.14969 28869295 PMC5641223

[acel13337-bib-0021] Lai, R. C. , Tan, S. S. , Yeo, R. W. Y. , Choo, A. B. H. , Reiner, A. T. , Su, Y. , Shen, Y. , Fu, Z. , Alexander, L. , Sze, S. K. , & Lim, S. K. (2016). MSC secretes at least 3 EV types each with a unique permutation of membrane lipid, protein and RNA. Journal of Extracellular Vesicles, 5, 29828. 10.3402/jev.v5.29828 26928672 PMC4770866

[acel13337-bib-0022] Lavasani, M. , Robinson, A. R. , Lu, A. , Song, M. , Feduska, J. M. , Ahani, B. , Tilstra, J. S. , Feldman, C. H. , Robbins, P. D. , Niedernhofer, L. J. , & Huard, J. (2012). Muscle‐derived stem/progenitor cell dysfunction limits healthspan and lifespan in a murine progeria model. Nature Communications, 3, 608. 10.1038/ncomms1611 PMC3272577 22215083

[acel13337-bib-0023] LeBrasseur, N. K. , Tchkonia, T. & Kirkland, J. L. (2015). Cellular senescence and the biology of aging, disease, and frailty. Nestle Nutrition Institution Workshop Series, 83, 11–18. 10.1159/000382054 PMC4780350 26485647

[acel13337-bib-0024] Livak, K. J. & Schmittgen, T. D. (2001). Analysis of relative gene expression data using real‐time quantitative PCR and the 2(‐Delta Delta C(T)) method. Methods, 25(4), 402–408. 11846609 10.1006/meth.2001.1262

[acel13337-bib-0025] Niedernhofer, L. J. & Robbins, P. D. (2018). Senotherapeutics for healthy ageing. Nature Reviews Drug Discovery, 17, 377. 10.1038/nrd.2018.44 29651106

[acel13337-bib-0026] Ogrodnik, M. , Miwa, S. , Tchkonia, T. , Tiniakos, D. , Wilson, C. L. , Lahat, A. , Day, C. P. , Burt, A. , Palmer, A. , Anstee, Q. M. , Grellscheid, S. N. , Hoeijmakers, J. H. J. , Barnhoorn, S. , Mann, D. A. , Bird, T. G. , Vermeij, W. P. , Kirkland, J. L. , Passos, J. F. , von Zglinicki, T. , & Jurk, D. (2017). Cellular senescence drives age‐dependent hepatic steatosis. Nature Communications, 8, 15691. 10.1038/ncomms15691 PMC5474745 28608850

[acel13337-bib-0027] Palmer, A. K. , Tchkonia, T. , LeBrasseur, N. K. , Chini, E. N. , Xu, M. , & Kirkland, J. L. (2015). Cellular senescence in type 2 diabetes: A therapeutic opportunity. Diabetes, 64, 2289–2298. 10.2337/db14-1820 26106186 PMC4477358

[acel13337-bib-0028] Pathan, M. , Fonseka, P. , Chitti, S. V. , Kang, T. , Sanwlani, R. , Van Deun, J. , Hendrix, A. & Mathivanan, S. (2019). Vesiclepedia 2019: A compendium of RNA, proteins, lipids and metabolites in extracellular vesicles. Nucleic Acids Research, 47, D516–D519. 30395310 10.1093/nar/gky1029 PMC6323905

[acel13337-bib-0029] Robinson, A. R. , Yousefzadeh, M. J. , Rozgaja, T. A. , Wang, J. , Li, X. , Tilstra, J. S. , Feldman, C. H. , Gregg, S. Q. , Johnson, C. H. , Skoda, E. M. , Frantz, M.‐C. , Bell‐Temin, H. , Pope‐Varsalona, H. , Gurkar, A. U. , Nasto, L. A. , Robinson, R. A. S. , Fuhrmann‐Stroissnigg, H. , Czerwinska, J. , & McGowan, S. J. … Niedernhofer, L. J. (2018). Spontaneous DNA damage to the nuclear genome promotes senescence, redox imbalance and aging. Redox Biology, 259–273. 10.1016/j.redox.2018.04.007 29747066 PMC6006678

[acel13337-bib-0030] Roos, C. M. , Zhang, B. , Palmer, A. K. , Ogrodnik, M. B. , Pirtskhalava, T. , Thalji, N. M. , Hagler, M. , Jurk, D. , Smith, L. A. , Casaclang‐Verzosa, G. , Zhu, Y. , Schafer, M. J. , Tchkonia, T. , Kirkland, J. L. , & Miller, J. D. (2016). Chronic senolytic treatment alleviates established vasomotor dysfunction in aged or atherosclerotic mice. Aging Cell, 15(5), 973–977. 10.1111/acel.12458 26864908 PMC5013022

[acel13337-bib-0031] Schafer, M. J. , White, T. A. , Iijima, K. , Haak, A. J. , Ligresti, G. , Atkinson, E. J. , Oberg, A. L. , Birch, J. , Salmonowicz, H. , Zhu, Y. , Mazula, D. L. , Brooks, R. W. , Fuhrmann‐Stroissnigg, H. , Tamar Pirtskhalava, Y. S. , Prakash, T. T. , Robbins, P. D. , Aubry, M. C. , Passos, J. F. , & LeBrasseur, N. K. (2017). Cellular senescence mediates fibrotic pulmonary disease. Nature Communications, 8, 14532. 10.1038/ncomms14532 PMC5331226 28230051

[acel13337-bib-0032] Shannon, P. , Markiel, A. , Ozier, O. , Baliga, N. S. , Wang, J. T. , Ramage, D. , Amin, N. , & Schwikowski, B. , & Ideker, T. (2003). Cytoscape: A software environment for integrated models of biomolecular interaction networks. Genome Research, 13, 2498–2504. 10.1101/gr.1239303 14597658 PMC403769

[acel13337-bib-0033] Tan, C. , Lai, R. , Wong, W. , Dan, Y. , Lim, S.‐K. , & Ho, H. (2014). Mesenchymal stem cell‐derived exosomes promote hepatic regeneration in drug‐induced liver injury models. Stem Cell Research & Therapy, 5, 76. 10.1186/scrt465 24915963 PMC4229780

[acel13337-bib-0034] Tazearslan, C. , Huang, J. , Barzilai, N. , & Suh, Y. (2011). Impaired IGF1R signaling in cells expressing longevity‐associated human IGF1R alleles. Aging Cell, 10, 551–554. 10.1111/j.1474-9726.2011.00697.x 21388493 PMC3094477

[acel13337-bib-0035] Tchkonia, T. , Zhu, Y. , van Deursen, J. , Campisi, J. , & Kirkland, J. L. (2013). Cellular senescence and the senescent secretory phenotype: therapeutic opportunities. Journal of Clinical Investigation, 123, 966–972. 10.1172/JCI64098 PMC3582125 23454759

[acel13337-bib-0036] Toh, W. S. , Lai, R. C. , Zhang, B. , & Lim, S. K. (2018). MSC exosome works through a protein‐based mechanism of action. Biochemical Society Transactions, 46, 843–853. 10.1042/BST20180079 29986939 PMC6103455

[acel13337-bib-0037] Toh, W. S. , Zhang, B. , Lai, R. C. , & Lim, S. K. (2018). Immune regulatory targets of mesenchymal stromal cell exosomes/small extracellular vesicles in tissue regeneration. Cytotherapy, 20, 1419–1426. 10.1016/j.jcyt.2018.09.008 30352735

[acel13337-bib-0038] Tran, D. , Bergholz, J. , Zhang, H. , He, H. , Wang, Y. , Zhang, Y. , Zhang, Y. , Li, Q. , Kirkland, J. L. , & Xiao, Z. X. (2014). Insulin‐like growth factor‐1 regulates the SIRT1‐p53 pathway in cellular senescence. Aging Cell, 13, 669–678. 25070626 10.1111/acel.12219 PMC4118446

[acel13337-bib-0039] Wu, D. & Prives, C. (2018). Relevance of the p53‐MDM2 axis to aging. Cell Death and Differentiation, 25, 169–179. 10.1038/cdd.2017.187 29192902 PMC5729541

[acel13337-bib-0040] Yoshida, M. , Satoh, A. , Lin, J. B. , Mills, K. F. , Sasaki, Y. O. , Rensing, N. , Wong, M. , Apte, R. S. , & Imai, S. I. (2019). Extracellular vesicle‐contained eNAMPT delays aging and extends lifespan in mice. Cell Metabolism, 30, 329–342.e5. 10.1016/j.cmet.2019.05.015 31204283 PMC6687560

[acel13337-bib-0041] Yousefzadeh, M. J. , Wilkinson, J. E. , Hughes, B. , Gadela, N. , Ladiges, W. C. , Vo, N. , Niedernhofer, L. J. , Huffman, D. M. , & Robbins, P. D. (2020). Heterochronic parabiosis regulates the extent of cellular senescence in multiple tissues. Geroscience, 42, 951–961. 32285290 10.1007/s11357-020-00185-1 PMC7286998

[acel13337-bib-0042] Zhang, B. , Yeo, R. W. Y. , Lai, R. C. , Sim, E. W. K. , Chin, K. C. , & Lim, S. K. (2018). Mesenchymal stromal cell exosome‐enhanced regulatory T‐cell production through an antigen‐presenting cell‐mediated pathway. Cytotherapy, 20, 687–696. 10.1016/j.jcyt.2018.02.372 29622483

[acel13337-bib-0043] Zhang, S. , Chu, W. C. , Lai, R. C. , Lim, S. K. , Hui, J. , & Toh, W. S. (2016). Exosomes derived from human embryonic mesenchymal stem cells promote osteochondral regeneration. Osteoarthritis Cartilage, 24, 2135–2140. 10.1016/j.joca.2016.06.022 27390028

[acel13337-bib-0044] Zhang, S. , Chuah, S. J. , Lai, R. C. , Hui, J. H. P. , Lim, S. K. , & Toh, W. S. (2018). MSC exosomes mediate cartilage repair by enhancing proliferation, attenuating apoptosis and modulating immune reactivity. Biomaterials, 156, 16–27. 10.1016/j.biomaterials.2017.11.028 29182933

[acel13337-bib-0045] Zhu, Y. I. , Tchkonia, T. , Pirtskhalava, T. , Gower, A. C. , Ding, H. , Giorgadze, N. , & Kirkland, J. L. (2015). The Achilles’ heel of senescent cells: From transcriptome to senolytic drugs. Aging Cell, 14, 644–658. 10.1111/acel.12344 25754370 PMC4531078

