# Supplementary material for: Mesenchymal stem cell‐derived extracellular vesicles reduce senescence and extend health span in mouse models of aging
Source: Aging Cell. 2021 Mar 16;20(4):e13337. doi: 10.1111/acel.13337 (PMC8045949; doi:10.1111/acel.13337)
Supplement: Supplementary file 1 — Fig S1‐6 [file ACEL-20-e13337-s002.pptx]

## Slide 1
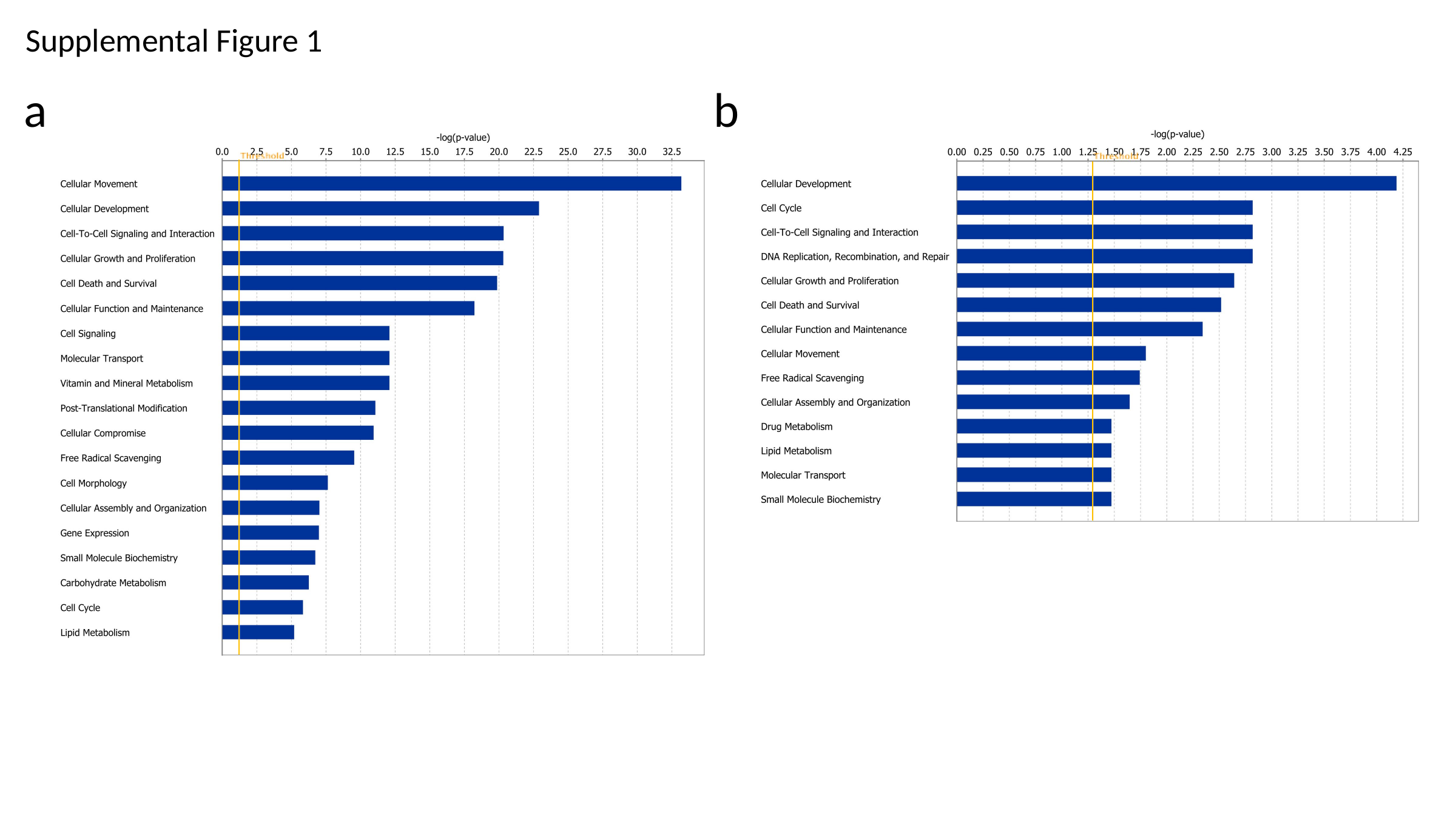

Supplemental Figure 1
a
b

## Slide 2
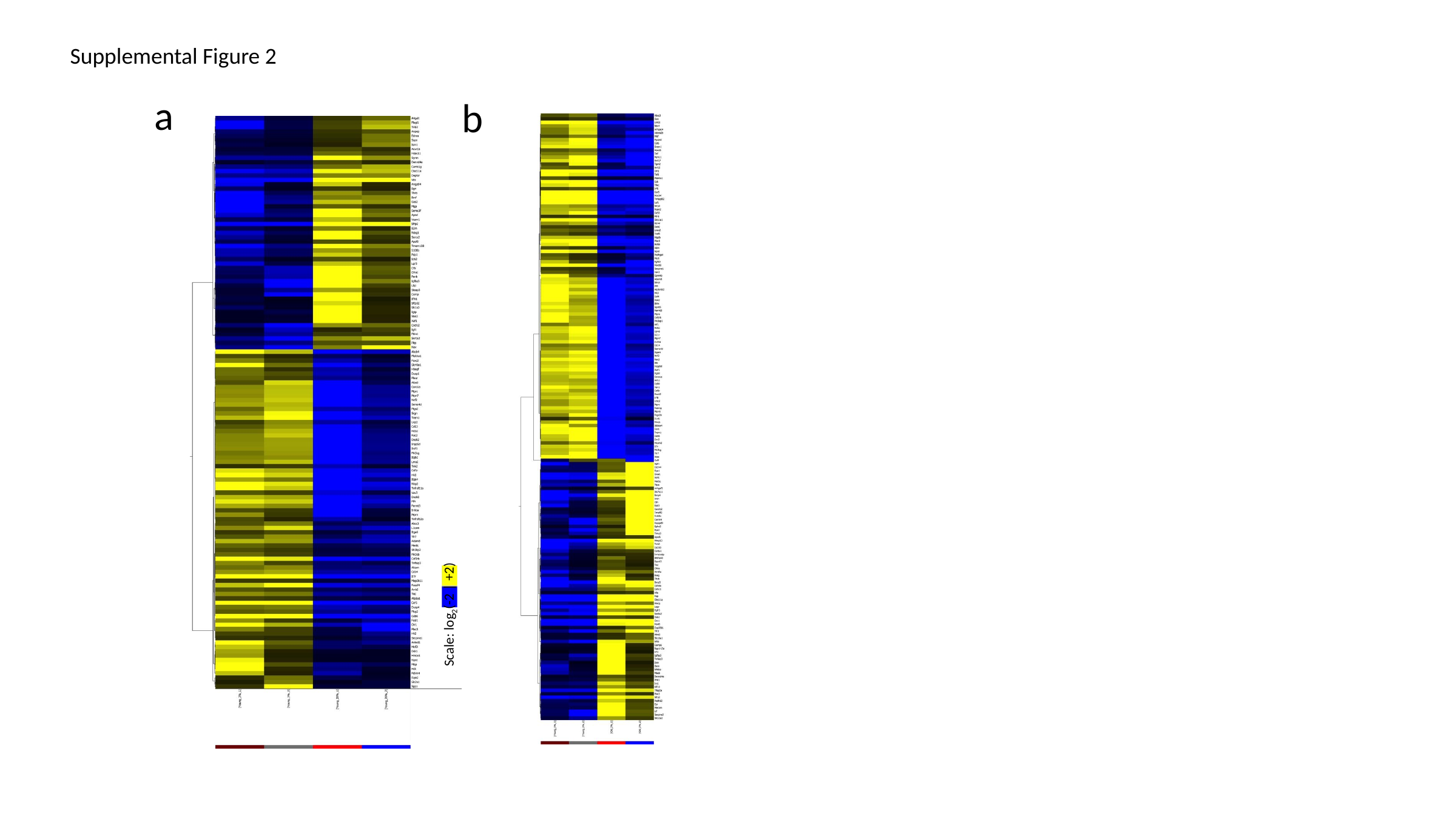

Supplemental Figure 2
a
b
 Scale: log2(-2 | +2)

## Slide 3
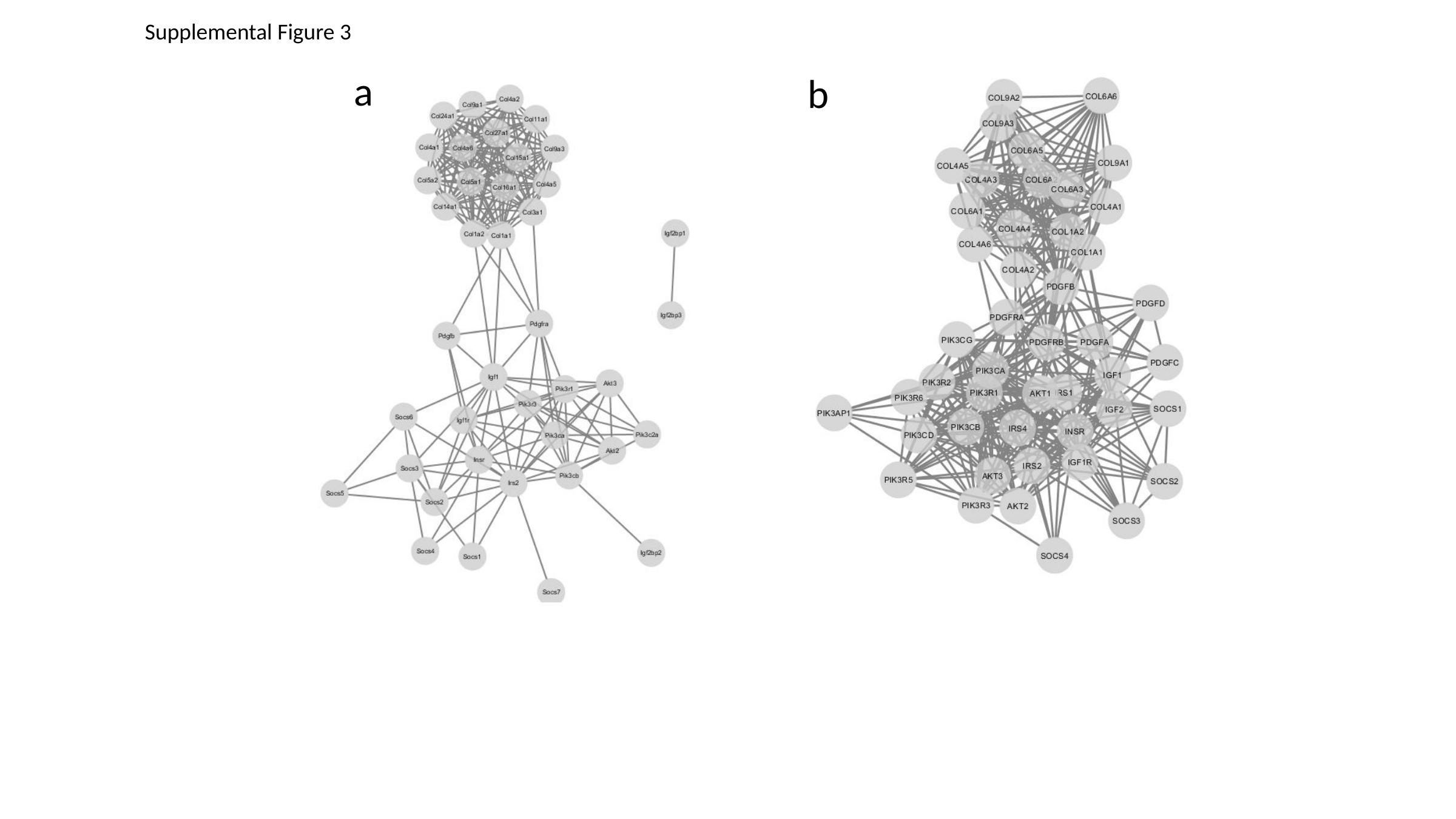

Supplemental Figure 3
a
b

## Slide 4
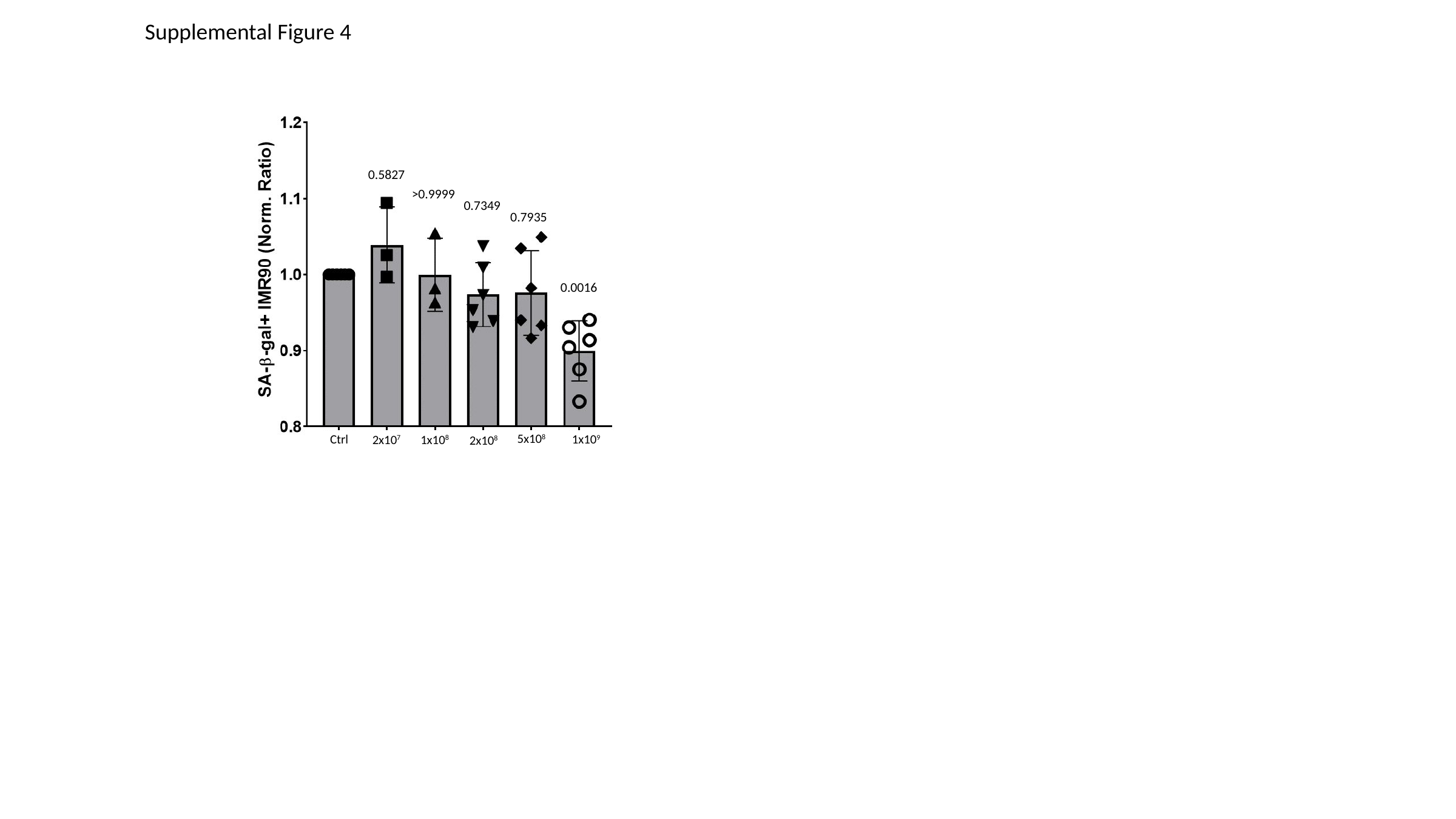

Supplemental Figure 4
0.5827
>0.9999
0.7349
0.7935
0.0016
5x10^8^
Ctrl
1x10^9^
2x10^7^
1x10^8^
2x10^8^

## Slide 5
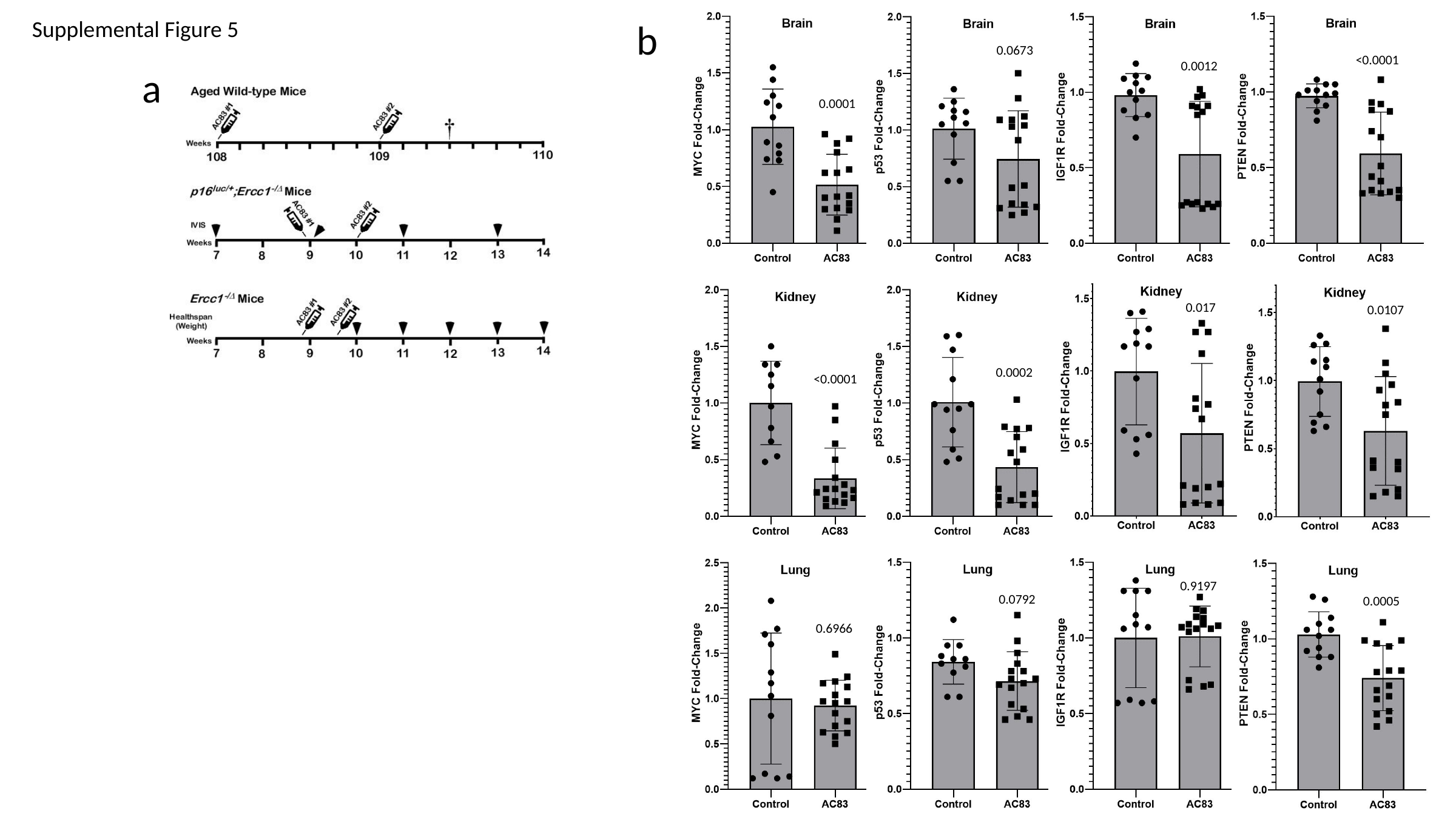

0.0673
<0.0001
0.0012
0.0001
0.017
0.0107
0.0002
<0.0001
0.9197
0.0792
0.0005
0.6966
Supplemental Figure 5
b
a

## Slide 6
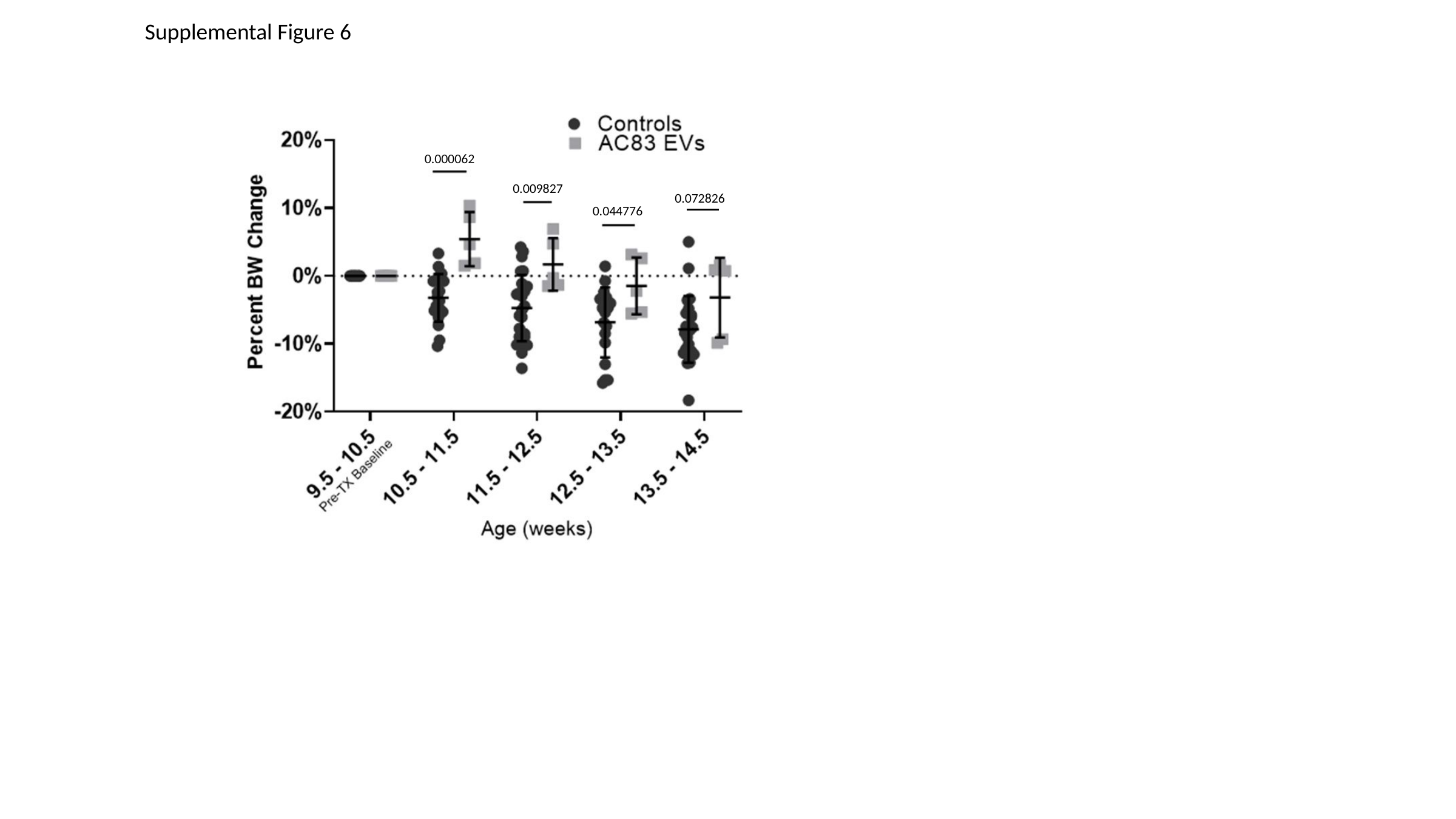

Supplemental Figure 6
0.000062
0.009827
0.072826
0.044776
